# Lymphangiogenic responses of lymphatic endothelial cells to steady direct-current electric fields

**DOI:** 10.1080/19336918.2023.2271260

**Published:** 2023-10-27

**Authors:** Linbo Guan, Ping Fan, Yufeng Wang, Xinghui Liu, Rui Liu, Wandi Ma, Huai Bai

**Affiliations:** a Laboratory of Genetic Disease and Perinatal Medicine, Key Laboratory of Birth Defects and Related Diseases of Women and Children of the Ministry of Education, West China Second University Hospital, Sichuan University, Chengdu, China; b Department of Obstetrics and Gynecology, West China Second University Hospital, Sichuan University, Chengdu, China; c Division of Peptides Related with Human Disease, West China Hospital, Sichuan University, Chengdu, China

**Keywords:** Alignment, cell migration, electrical stimulation, lymphangiogenesis, lymphatic endothelial cells, orientation, signal transduction

## Abstract

Lymphangiogenesis plays pivotal roles in diverse physiological and pathological conditions. Steady direct-current electric fields (DC EFs) induce vascular endothelial behaviors related to angiogenesis have been observed. This study investigated the effects of DC EFs on the lymphangiogenic response of lymphatic endothelial cells (LECs). We demonstrated that EFs stimulation induced directional migration, reorientation, and elongation of human LECs in culture. These lymphangiogenic responses required VEGF receptor 3 (VEGFR-3) activation and were mediated through the PI3K-Akt, Erk1/2, and p38 MAPK signaling pathways in relation to the reorganization of the actin cytoskeleton. Our results indicate that endogenous EFs may play a role in lymphangiogenesis in vivo, and VEGFR-3 signaling activation may be involved in the cellular function of LECs driven by EFs.

## Introduction

The lymphatic vessels regulate tissue fluid homeostasis, immune cell transport, and intestinal lipids [1]. The formation of new lymphatic vessels (that is, lymphangiogenesis) occurs at a high level during embryogenesis. During embryonic development, lymphatic progenitor cells form new lymphatic vessels through cell differentiation, proliferation, and migration [2]. In adults, lymphangiogenesis is reactivated in pathological conditions such as wound healing, lymphedema, inflammation, and the presence of tumors [3]. Individuals with dysfunctional lymphatic vessels often suffer from various diseases including weakened immune responses, impaired metabolic function, tissue swelling, fibrosis, inflammation, and lymphedema [4]. The stimulation of new lymphatic vessel formation can prevent or reverse the progression of these abnormalities. However, lymphangiogenesis is relatively understudied and stimulating therapeutic lymphangiogenesis poses challenges to the precise control of lymphatic vessel formation [5].

Vascular endothelial growth factor-3 (VEGFR-3) is a member of the receptor tyrosine kinase family and is widely expressed in vascular endothelial cells during embryonic development, but becomes strongly restricted to lymphatic endothelial cells (LECs) in the adult organism under physiological conditions. VEGFR-3 plays important roles both in lymphangiogenesis and angiogenesis. Upon stimulation by its ligand, VEGF-C, VEGFR-3 can form both homodimers and heterodimers with VEGFR-2 and activate several downstream signaling pathways, including Erk1/2 and Akt. Despite certain similarities with VEGFR-2, the molecular features of VEGFR-3 signaling remain largely unknown. VEGF-C activates Akt signaling through the formation of the VEGFR-3/VEGFR-2 complex, whereas Erk is activated by the VEGFR-3 homodimer. NRP1 and VE-PTP are involved in regulating VEGFR-3 signaling. Functionally, the Erk and Akt pathways are important for the proliferation, migration, and survival of LECs.

Endogenous electric fields (EFs) are detected during embryonic development and wound healing, when active vascular vessel formation occurs. These physiological EFs play important roles in both physiological and pathological processes, such as embryonic development, wound healing, and tumor invasion and metastasis [6,7], in which EFs can be implicated in instructing cells with directional or positional information [8–10]. In vitro studies have shown that small EFs regulate many crucial cellular behaviors, such as cell migration, cell proliferation, and cell differentiation [7,11–13]. Substantial evidence has shown the potential of EFs to direct and enhance the regrowth of damaged epithelial wounds [14]. Electrical stimulation has emerged as a potentially novel approach for inducing angiogenesis in vivo [15–18]. Our recent studies demonstrated that EF can induce angiogenic responses in vascular endothelial cells, such as directional migration, reorientation, elongation, and tube-like structure formation [18–22].

Lymphatic capillaries (initial lymphatics) consist of a single layer of partially overlapping thin LECs. Lymphangiogenesis, including angiogenesis, is a complex process involving the formation of new lymphatic vessels from preexisting lymphatic vessels based on the capacity of LECs to migrate, proliferate, elongate, and form vessel structures. One possibility shown by studies on vascular endothelial cells is that EF stimulation may affect LECs and contribute to the formation of new lymphatic vessels. A previous study demonstrated that low-frequency electric current stimulation can be used to treat patients with lymphedema, suggesting that physical stimuli could have an important influence on lymphatic function [23]. However, despite extensive studies on the mechanisms of action of lymphangiogenic factors at the molecular level [3], little is known about how biophysical stimuli affect lymphatic function. To date, only one study has reported that pulsed-direct-current (DC) EF stimulation promotes the proliferation and migration of LECs, and that electro-stimulation activates lymphatic function by activating p38 MAPK [24]. Systemic studies on the effects of a steady DC EF at a physiological magnitude on LECs have not been conducted.

In this study, we hypothesized that EF regulates the behavior of human LECs during lymphangiogenesis. We examined the effects of EFs on LEC migration, elongation, and orientation, as EF may be an important signal for inducing cellular behaviors related to lymphangiogenesis. We also determined that VEGFR-3 and its downstream signaling pathways, such as PI3K/Akt and MAPKs, mediate lymphangiogenic effects. Our results indicated that EF augmented the migration rate of human LECs (HLECs) and induced cell anodal migration, reorientation, and elongation. The underlying mechanism related to EF-mediated cell migration velocity and elongation response may involve the activation of VEGFR-3 and its downstream signaling cascade.

## Materials and methods

### Cell cultures and reagents

HLECs from the ScienCell (ScienCell, Carlsbad, CA, USA) (catalog #2500) was used. HLECs were maintained in complete endothelial cell medium (ECM), which consisted of 500 mL of basal medium, 25 mL of fetal bovine serum (FBS), 5 mL of endothelial cell growth supplement (ECGS), and 5 mL of penicillin/streptomycin solution (P/S). Primary antibodies against Akt, Erk1/2, p38 MAPKs (pan-specific antibody and active form), and GAPDH were purchased from Cell Signaling Technology (Danvers, MA, USA). Akt inhibitor MK-2206 2HCL, Erk1/2 inhibitor U0126, p38 inhibitor SB203580, and VEGFR-3 inhibitor SAR131675 were obtained from Selleck Chemicals (Selleck, USA). DyeLight 680-labeled secondary antibody against rabbit IgG (H+L) was purchased from PKL, Inc. Bicinchoninic acid (BCA) Protein Assay Kits were purchased from Thermo Inc. A whole-protein extraction kit was purchased from KeyGEN Biotech.

### Electrotaxis stimulation

HLECs were cultured in complete ECM. The experimental set-up and EF exposure protocols were similar to those reported previously [25] (Fig. S1). Briefly, HLECs at a density of approximately 3 × 10^4^ cells/mL for morphological analysis or 1 × 10^5^ cells/mL for protein analysis were seeded into specially made troughs formed by two parallel (2 cm apart) strips of glass coverslips (No. 1, length 22 mm or 50 mm) fixed to the base of the dish with silicone grease (Dow Corning, DC4). Cells were incubated for 24–48 h (37°C, 5% CO_2_), allowing them to settle and adhere to the base of the dish before the roof of a No. 1 coverslip was applied and sealed with silicone grease. The final dimensions of the chamber through which the current was passed were 22 × 10 × 0.2 mm or 50 × 10 × 0.2 mm. Agar-salt bridges not less than 15 cm long were used to connect silver/silver-chloride electrodes in beakers of Steinberg’s solution (58 mM NaCl, 0.67 mM KCl, 0.44 mM Ca(NO_3_)_2_, 1.3 mM MgSO_4_, 4.6 mM Trizma base, pH 7.8–8.0) to pools of excess culture medium on either side of the chamber. This prevented diffusion of the electrode products into the culture medium. EFs in the physiological range of 100–300 mV/mm were used. The field strength was measured directly at the beginning, end, and during each experiment. No fluctuations in field strength were observed. A series of images were taken with an image analyzer immediately before EF exposure and at 4, 8, 12, and 24 h after EF exposure [25]. Time-lapse imaging was performed to record the cell migration using a time-lapse microscope (Nikon Ti-E). Cell migration was recorded for 4 h by capturing images every 5 min during the recording period. For drug inhibition experiments, cells were incubated with the Akt inhibitor MK-2206 2HCL (10 μM), Erk1/2 inhibitor U0126 (20 μM), p38 inhibitor SB203580 (50 μM), and VEGFR-3 inhibitor SAR131675 (1 μM) for 1 h before EF stimulation. The same drug concentration was used during EF exposure in a CO_2_ incubator.

### Quantification of cell behavior

The time-lapse images or serial pictures were analyzed using Image-Pro Plus software. Cell migration was quantified as described previously reported method [25,26]. Cell migration velocity was calculated from the full distance of cell migration at a given time. Cell orientation was quantified using the orientation index (Oi), as described in detail in previous publications [18,25] (Fig S1). Cell elongation was quantified by calculating the long:short axis ratio of the cells using a previously reported method [18]. To quantify cell behavior, cells from three independent experiments were analyzed.

### Immunofluorescent staining for filamentous actin (F-actin)

Confocal microscopy was performed to examine the organization of F-actin in cells as described previously [27]. Briefly, EF-stimulated HLECs or unstimulated control cells were washed thrice with phosphate-buffered saline (PBS), fixed for 5 min in 4% paraformaldehyde (PFA), washed extensively in PBS, and permeabilized with 0.1% Triton X-100. After three washes with PBS, the cells were incubated with conjugated DyLight 488 phalloidin (1:40) for 40 min at room temperature and washed three times. The cells were immediately viewed under a confocal laser scanning microscope (CLSM).

### Analysis of the cellular F/G-actin ratio

For immunofluorescence of HLECs, F-actin was labeled with Alexa Fluor 593 phalloidin(1:200; KTC4009, Abbkine), and G-actin was labeled with Alexa Fluor 488 deoxyribonuclease I (0.3 μM; D12371, Invitrogen) for 1 h at room temperature.

For western blotting, we used the G-actin/F-actin assay kit (Cat. #BK037; Cytoskeleton Inc., USA) according to the manufacturer’s protocol. Briefly, EF-stimulated HLECs or unstimulated control cells were lysed in lysis buffer (50 mM PIPES pH 6.9, 50 mM NaCl, 5 mM MgCl_2_, 5 mM EGTA, 5% (v/v) glycerol, 0.1% NP-40, 0.1% Triton X-100, 0.1% Tween-20, and 0.1% 2-mercaptoethanol) freshly supplemented with 100 mM ATP and 1× protease inhibitor before use. Aliquots of lysates containing equal amounts of cellular proteins were centrifuged at 2000 rpm for 15 min to eliminate cell debris, and the supernatant fraction from each sample was subjected to high-speed centrifugation (100,000 × g in a TLA100.3 rotor, Beckman Coulter Inc.) for 1 h. The resulting supernatant (G-actin) and pellet (F-actin) fractions were examined for actin using western blot analysis. Actin signals in the G- and F-actin fractions were quantified using the Image J software to determine the F/G-actin ratio. The relative F/G-actin ratios were calculated by normalizing the ratios of control samples.

### *Analysis of phosphorylation of* VEGFR-3

To determine the activity of VEGFR-3, Immunofluorescence staining and enzyme-linked immunosorbent assay (ELISA) for phosphorylated VEGFR-3 have been used.

EF stimulation procedures for HLECs were similar to those described previously. After EF stimulation of the HLECs, the PBS washed cells were fixed in 4% PFA for 10 min at 37°C followed by permeabilization with 0.1% Triton X-100 for 10 min at room temperature and blocked with 3% bovine serum albumin (BSA) for 1 h at 37°C. The fixed cellular structures were incubated with rabbit anti-VEGFR-3, directed against the total (dilution 1:200) or the active (phosphorylated, dilution 1:200) form of the proteins (Affinity Biosciences or Abcam Inc.) for 1 h at 37°C followed by a similar incubation with a secondary goat anti-rabbit DyeLight 680-labeled antibody (1:1000, KPL) as above. The cells were stained with 4’,6-diamidino-2-phenylindole DAPI (Invitrogen) and mounted using Vectashield (Vector Laboratories, Burlingame, CA, USA). The images were obtained using an Olympus confocal microscope FV1000 (Olympus, Tokyo, Japan). In addition, phosphorylated VEGFR-3 in the cell lysates was measured using the RayBio Human Phosphotyrosine VEGFR-3 ELISA Kit (Cat# PEL-VEGFR-3-Y; RayBiotech, Inc., USA), according to the manufacturer’s protocol.

### Western blot analysis

HLECs were treated with EF (200 mV/mm) for 5, 10, 15, 30, and 60 min. After each time point, the medium was aspirated, and cells were lysed in 100 μL of lysis buffer (20 mM Tris-HCl, 10% glycerol, 0.2 mM EDTA, 0.137 M NaCl, 1% NP-40) supplemented with a complete protease and phosphatase inhibitor cocktail (Roche). The cellular extract was incubated for 30 min on ice and then subjected to centrifugation at 12,000 × g for 15 min at 4°C. The supernatant was collected, and the amount of protein in each sample was quantified using a BCA colorimetric assay with BSA as a standard. Samples containing 50 μg of total protein were loaded onto 10% Sodium dodecyl-sulfate (SDS)-polyacrylamide gels, and the electrophoresed samples were transferred onto polyvinylidene fluoride membranes, as previously described [28]. Individual blots were incubated at 4°C overnight with rabbit polyclonal antibodies against Akt (9272; 1:1000; Cell Signaling Technology, Danvers, MA), pAKT (4063; 1:1000; Cell Signaling Technology), Erk (9102; 1:1000; Cell Signaling Technology), pErk (4377; 1:1000; Cell Signaling Technology), p38 (9212; 1:1000; Cell Signaling Technology), and p-p38 (9211; 1:1000; Cell Signaling Technology), followed by incubation with a 1:10000 dilution of fluorescently labeled goat anti-rabbit IgG antibody (072-06-15-16; KPL, Gaithersburg, MD). The intensity of the bands on the western blots was quantified using Quantity One software (Bio-Rad).

### Statistical analysis

Results are expressed as means ± standard deviation (SD). Values from the groups were compared using an unpaired two-tailed Student’s t-test or Duncan’s test. Statistical significance was set at P-value <.05 significant.

## Results

### Small EFs direct migration of LECs toward the anode

**Figure 1.**
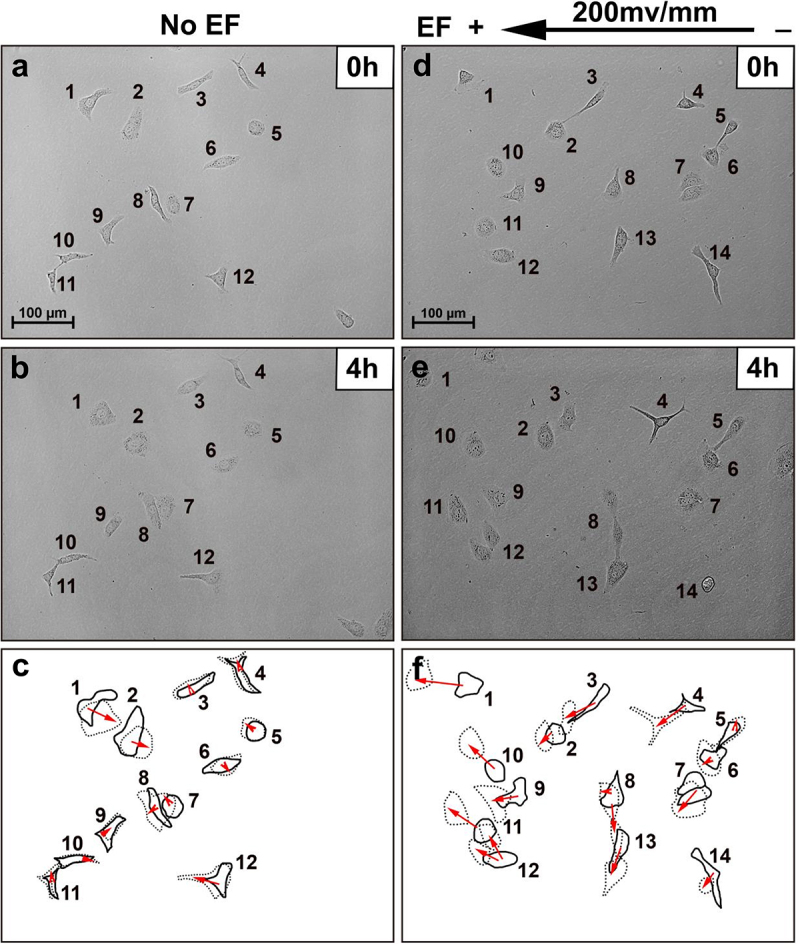
HLECs migrate toward the anode in small physiological EFs. (a, b) random migration of cells over a 4 h period without EF stimulation. (d, e) cells migrated to the anode left in response to EF stimulation over 4 h (see Supplementary video S1). Cells are identified by numbering. Bottom panels (c, f) show the outlines of the cells at the beginning and end of each experiment, with migration direction indicated by the arrows. Scale bar = 100 µM.

**Figure 2.**
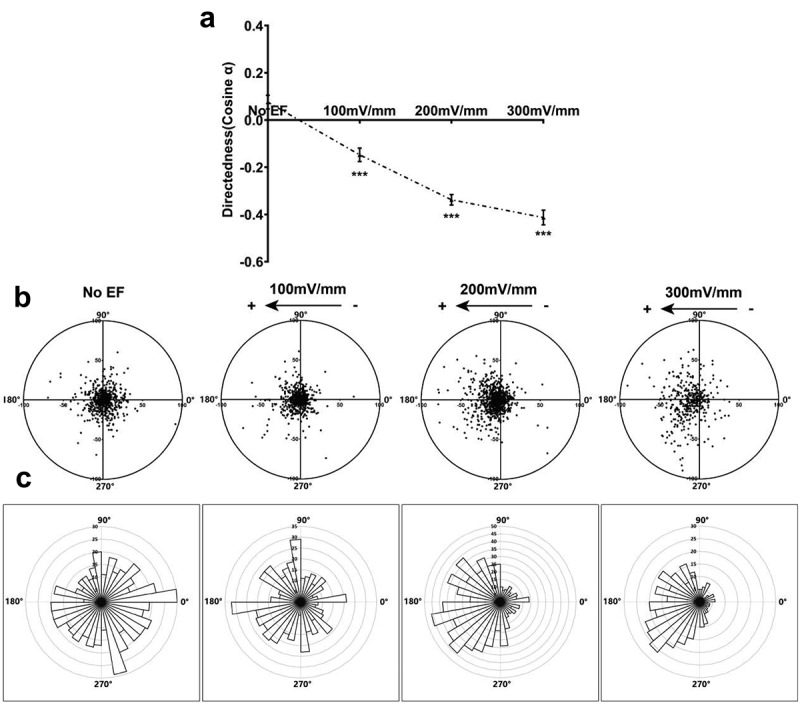
Anode-directed cell migration is voltage dependent. (a) directedness of HLECs in EFs. (b) migration of cells over a 4-h period. Each point represents the final position of a single cell relative to their starting position at 0 h at the origin. (c) circular histograms for cells in (b). Migrated HLECs show clear biased distribution toward the anode in EFs, indicating anode migration of cells. Distribution of migration angles were calculated from x–y coordinates at the beginning and end of the 4-h cell track. Angles are grouped in 10° intervals, with the radius of each wedge indicating numbers of cells. All data are shown as means ± SEM and representative of independent experiments from three individual preparations (375–787 cells tracked). ***P<.001, when compared with that in control with no EF (0 mV).

In the absence of an EF, HLECs moved randomly with an average cosine of 0.08 ± 0.03, while exposed to DC EFs (100, 200, or 300 mV/mm) (4 h) had an average cosine of −0.15 ± 0.03, −0.34 ± 0.02, and −0.41 ± 0.03, showing significantly motility enhancement and migrated steadily toward the anode (Figures 1, 2a, and Supplementary Video 1). With an increase in EF strength, the proportion of HLECs increased moving toward to anode gradually (Figure 2b,c).

**Figure 3.**
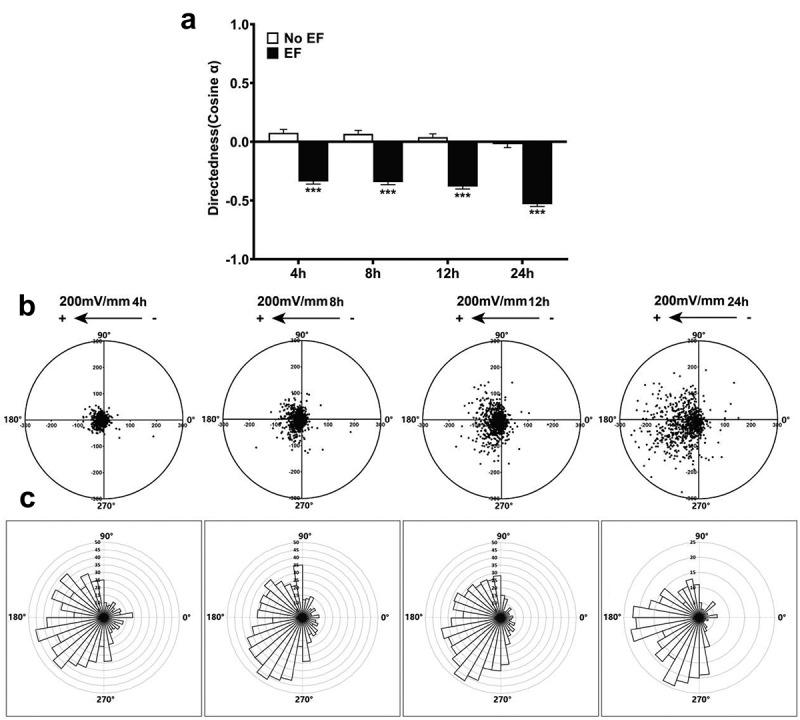
Anode-directed cell migration is time dependent. (a) directedness of HLECs at field strength of 200 mV/mm for 4, 8, 12, and 24 h. (b) migration of cells over different time periods. Each point represents the final position of a single cell relative to their starting position at 0 h at the origin. (c) circular histograms for cells in (b). Migrated HLECs show biased distribution toward the anode in EFs, indicating anode migration of cells. Distribution of migration angles at the beginning and the end of cell track. Angles are grouped in 10° intervals, with the radius of each wedge indicating numbers of cells. Results are means±SEM and representative of independent experiments from three individual preparations (603–787 cells tracked). ***P<.001, when compared with that in corresponding control with no EF (0 mV).

In addition, HLECs exposed to DC EFs (200 mV/mm) had an average cosine of −0.53 ± 0.02 within 24 h, which was significantly different from the control cells (p < .001). Of the cells, 73.82% moved toward the anode within 4 h, with 87.17% migrating anodally after 24 h at 200 mV/mm (Figure 3). Cells persisted in migrating toward the anode for over 24 h.

### Small EFs enhance migration speed

**Figure 4.**
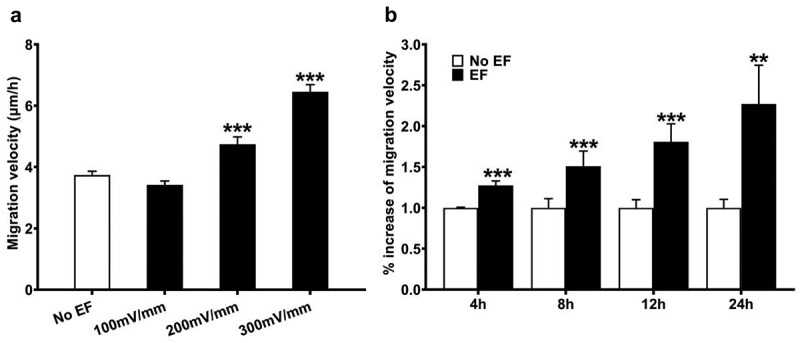
Small physiological EFs enhance migration speed of HLECs. (a) migration speed of HLECs increased with increasing EF strengths, or (b) when stimulation time was increased from 4 to 24 h (200 mV/mm). Results are mean±SEM from three independent experiments. ** *p* < .01, *** *p* < .001, when compared with that in corresponding control with no EF (0 mV).

HLECs moved randomly at a velocity of 3.74 ± 0.13 μM/h in the absent of EF. At the field strengths of 100, 200, and 300 mV/mm (4 h), the average cell migration velocities were 3.42 ± 0.13, 4.74 ± 0.24, and 6.46 ± 0.23 μM/h, respectively, showing voltage-dependent effect. The migration velocities of cells treated with 200 and 300 mV/mm showed a significantly increase compared to the no EF-treated control culture (both *p* < .001) (Figure 4a).

In addition, with an increase in stimulation time (4 h, 8 h, 12 h and 24 h) in the EF (200 mV/mm), the speed of the migrating cells also increased (p < .01, p < .001), indicating a time-dependent effect (Figure 4b).

### Small EFs dramatically reorient and elongate LECs

**Figure 5.**
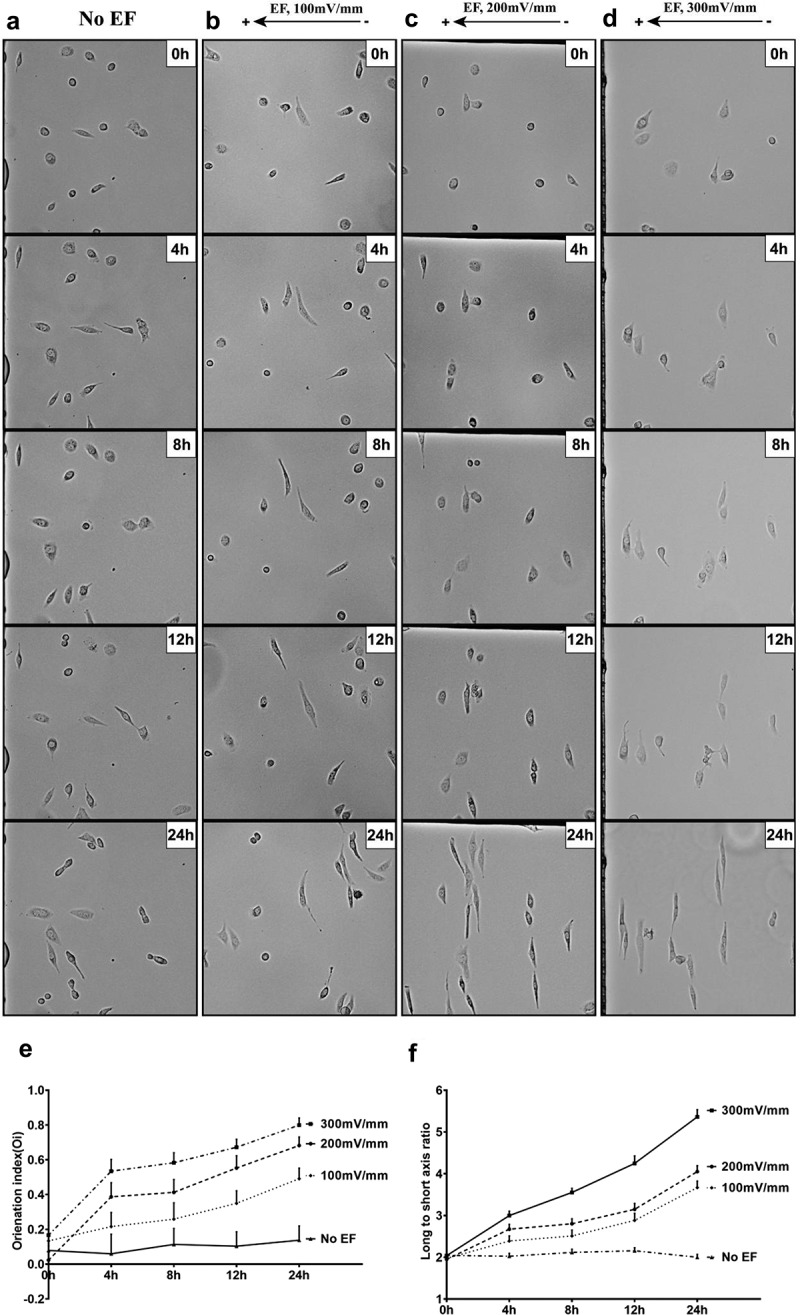
HLECs reoriented and elongated in a small physiological EF. HLECs exposed to small, applied EF (100, 200 and 300 mV/mm). Cells showed obvious perpendicular orientation and elongation (b, c, d), whereas control cells that were not subject to EF showed no responsiveness in typical morphology (a).Time and voltage dependency of orientation and elongation of HLECs in a small physiological EF. Orientation index (e) and elongation (f) as a function of time and voltage (*n*=31–129 from at least three independent experiments).

When cultured in EFs (100, 200, and 300 mV/mm), HLECs were aligned with their long axes perpendicular to the EF vector. The cellular response to EF increased with culture time, as shown in Figure 5. In addition, the cells appeared significantly elongated in the EF culture, whereas the control cells (no EF) showed a random alignment with typical endothelial morphology (Figure 5).

We further quantified cell alignment using the orientation index Oi=cos2(α). The cells cultured in an EF at strengths of 100, 200, and 300 mV/mm for 4 h, the values of orientation index (Oi) were 0.22 ± 0.08, 0.39 ± 0.08, and 0.53 ± 0.07, respectively. The Oi of the cells increased with an increase in the applied EFs, indicating a voltage dependence (Figure 5e). Compared to control cells (no EF), the orientation response of HLECs was also time-dependent in EFs, with a linear increase in Oi in EFs (4, 8, 12, and 24 h) (Figure 5e). EF stimulation at 300 mV/mm for 24 h, showed that almost all cells were perpendicular to the EF vector.

The cell elongation was quantified using the long-to-short axis ratio. A perfectly round cell has a long:short axis ratio of 1, and as the cell elongates, this ratio increases. Control cells (no EF) showed no increase in the long:short axis over 24 h of culture (Figure 5a, f). The long:short axis ratio of cells exposed to EFs showed gradual cell elongation throughout the 24 h experimental period (Figure 5b, c, d, f). The elongation responses were time and voltage dependent.

The morphology of the control and EF-treated cells stained with phalloidin-DyLight 488 (for staining F-actin) showed F-actin alignment with the EF-treated cells (Fig. S2). EF-stimulated cells showed an increase in the F-actin/G-actin ratio in IFA (Fig. S3A, B) and western blotting (Fig. S3 C, D).

### PI3K/Akt and MAPKs signaling are involved in the EF-induced responses of HLECs

Several intracellular signaling pathways are involved in the underlying mechanisms of lymphangiogenic responses. PI3K/AKT and MAPKs are critical molecules for these elements.

**Figure 6.**
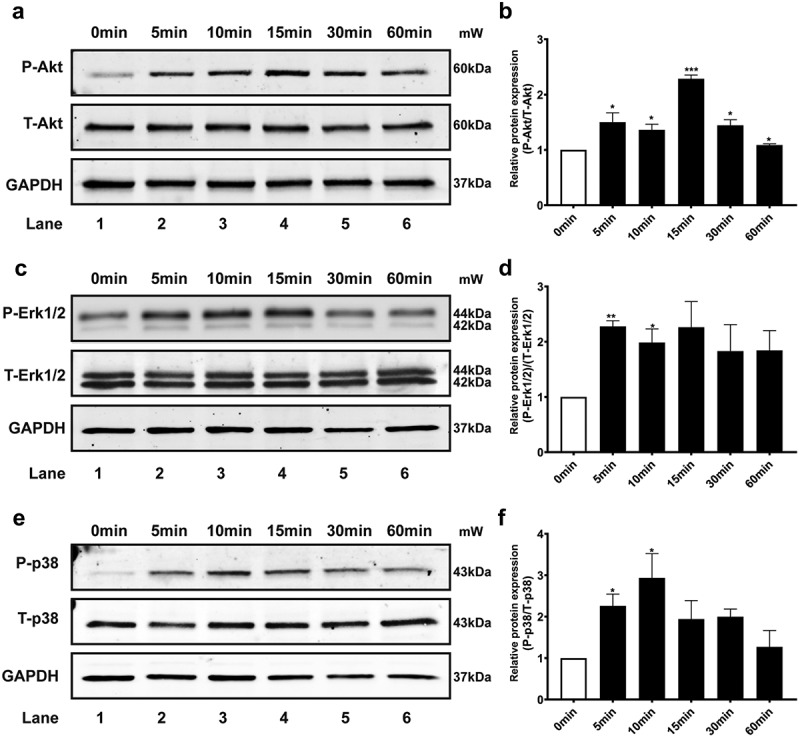
Effect of EF on activation of Akt, Erk1/2, and p38 MAPKs signaling pathways in HLECs. (a, c, and e) protein expression of Akt and Akt-phosphorylation, Erk1/2 and Erk1/2-phosphorylation, and p38 and p38-phosphorylation in different treatment groups (before or after EF stimulation) were detected using western blotting with anti-T-Akt and anti-p-Akt, anti-T-Erk1/2 and anti-p-Erk1/2, and anti-T-p38 and anti-p-p38. (b, d, and f) quantification of Akt, Erk1/2 and p38 MAPKs protein level. GAPDH was used as an internal control. Data are presented as the mean±SEM of at least three biological replicates, *p<.05, **P<.01, or ***P<.001, compared with control before EF treatment (0 min).

HLECs exposed to low applied EFs (200 mV/mm) showed prelymphangiogenic responses, including directional migration, elongation, and perpendicular orientation in the EF. Because the Akt and MAPKs signaling pathways have previously been shown to be involved in lymphangiogenesis, phospho-specific antibodies were used to measure the activation of Akt, Erk1/2, and p38 MAPKs after EF treatment. There was obvious early activation of Akt, Erk1/2, and p38 MAPKs at the 5 min time-point, as measured by elevated levels of Akt, Erk1/2, and p38 phosphorylation in HLECs (Figure 6), suggesting primary activation of these signaling pathways in response to EF stimulation.

To determine the specific role of Akt, Erk1/2, and p38 MAPKs activation in EF-mediated responses (migration velocity and directionality, as well as orientation and elongation) of HLECs, specific inhibitors of Akt (Akt-i, MK-2206 2HCL), Erk (Erk-i, U0126), and p38 (p38-i, SB203580) were used.

The results of the migration assay with EF treatment at 200 mV/mm demonstrated that cells treated with Akt, Erk1/2, and p38 inhibitors showed significant inhibition of cell migration compared to cells in EF culture without inhibitor treatment (Fig. S4A). In addition, Akt, Erk1/2, and p38 inhibitors completely abolished the elongation response of cells in EF when compared with cells in EF culture without inhibitor treatment (Fig. S4D). However, the inhibition of Akt, p38, or Erk1/2 activity did not significantly inhibit the directionality or alignment (reorientation) responses of HLECs in EF culture compared to cells in EF culture without inhibitor treatment (Fig. S4 B and C).

### Pre-lymphangiogenic responses of HLECs requires VEGFR-3 activation

VEGFR is the proximal element that transduces the chemical signal (VEGF-C) and induces lymphangiogenic responses. Among VEGFRs, VEGFR-3 plays a central role in the transition of LECs to lymphatic vessels (lymphangiogenesis).

**Figure 7.**
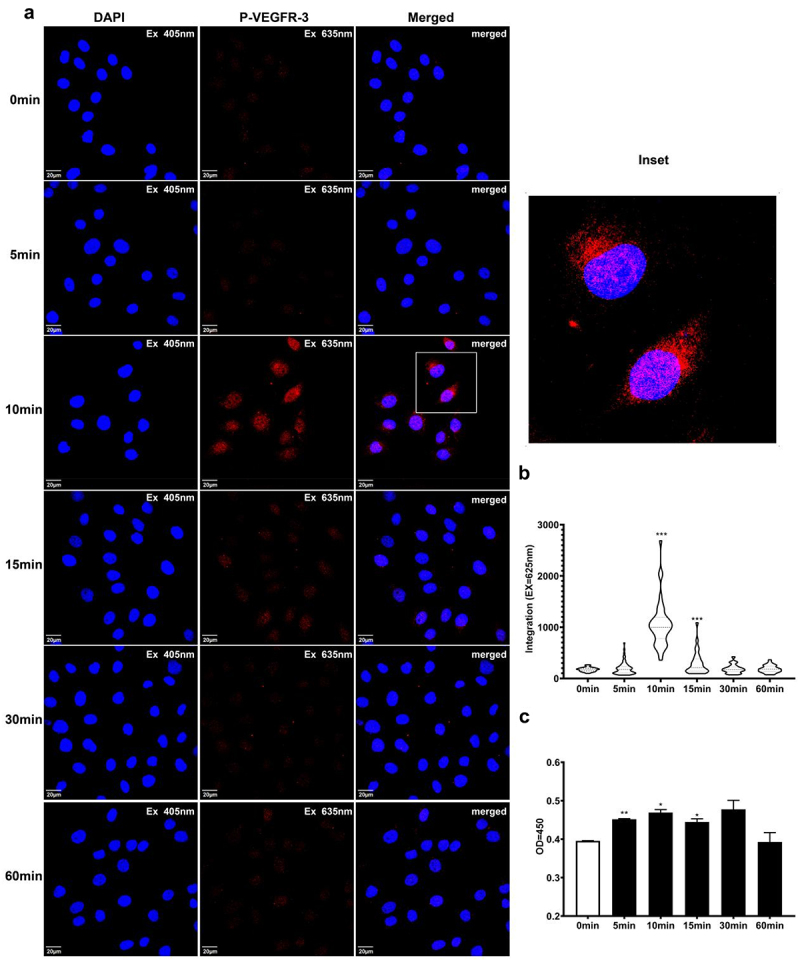
Time course of phosphor-VEGFR-3 activation of HLECs after application of EF (200 mV/mm). Antiphospho-VEGFR-3 was detected using immunofluorescence staining and ELISA (see methods). EF-treated HLECs demonstrated immediate VEGFR-3 phosphorylation within 10 min. Protein expression was quantified using CLSM and ELISA. The images show representative immunolabeled cellular structures (a). The histograms depict the relative immunofluorescence (b) and OD from cell lysate (c) of the phosphorylated protein. Data were from three separate experiments. The error bars represent the standard error (SE). *P< .05, **P< .01, ***P< .001, significantly different from the untreated control (0 minutes). Initial magnification of the images: ×200.

To elucidate the mechanisms employed by EF to regulate pre-lymphangiogenic responses, we explored the effects of EF on VEGFR-3 activity in HLECs. The p-VEGFR-3 levels in the cells were determined at the indicated time points (0, 5, 10, 15, 30, and 60 min) after exposure to EF (200 mV/mm). Immunofluorescence confocal microscopy revealed that EF exposure significantly enhanced VEGFR-3 phosphorylation. A marked elevation of p-VEGFR-3 in cells was observed as early as 10 min after the onset of EF; this gradually decreased from 10 to 15 min, and then further decreased after 30 min (Figure 7a,b). ELISA results further confirmed the IFA results (Figure 7c): EF-stimulated HLECs exhibited significant increase in phosphor-VEGFR-3 levels at 5 min, 10 min and 15 min (114%, 119% and 112%, respectively) compared to the no EF-treated control cells (0 min) (P = .001, 0.011 and 0.026, respectively).

Incubating HLECs with the specific VEGFR-3 inhibitor SAR131675 (1 µM) for 4 h significantly reduced the migration speed of the cells in EF (Fig. S5 A – C) (EF with the inhibitor vs EF without the inhibitor: 3.66 ± 0.15 μM/h vs 5.05 ± 0.19 μM/h; *P* < .001) (Fig. S5 D). The Long to Short axis ratio of the cells also decreased after the inhibition of VEGFR-3 activity, indicating that the inhibitor may abolish the EF-mediated effects on cytoskeletal stress fibers during field exposure (Fig. S5 C and G). Interestingly, inhibition of VEGFR-3 activity did not abrogate the directionality of the cell migration response to EF (Fig. S5 E), whereas significant Oi values remained (Fig. S5 F), indicating that different signaling mechanisms related to cellular responses may be involved (Fig S5 E and F).

In addition, inhibiting VEGFR-3 activity with a VEGFR-3 inhibitor inhibited the effects of EF on Akt, Erk 1/2, and p38 MAPKs activation, suggesting that the EF-mediated effects on these signaling pathways are dependent on VEGFR-3 (Fig. S6).

We have evaluated the effect of drugs used in our experiments. The results showed that there is no increase in p-VEGFR-3 (Fig. S7), and p-Akt, p-Erk 1/2 and p-p38 MAPKs (Fig. S8) with EF upon respective inhibitor treatment.

## Discussion

The lymphatic vasculature is an arborized network that runs parallel to the blood vasculature, and plays an important role in various physiological and pathological conditions. However, compared with the blood vascular system, this system has not been widely studied. While it has been proposed that electric stimulation affects vascular endothelial cells in vitro as well as in vivo, and may be a new approach to promoting angiogenesis in vivo [15,17], the present study focused on the EF-mediated effects of lymphangiogenic responses of LECs. We provide novel evidence that steady DC EFs affect LECs and cause pre-lymphangiogenic responses. These effects require VEGFR-3 activation. The PI3K-Akt, Erk1/2, and p38 MAPKs signaling pathways are involved.

Previous studies have demonstrated that electrical stimulation can induce significant changes in vascular endothelial cells. For example, DC EFs induce directional migration and increase cell migration rates, as well as the orientation and elongation of human umbilical vein endothelial cells (HUVECs) and human microvascular endothelial cells (HMEC-1s) [18,20]. Our results in LECs suggest that there could be direct effects of this type of electrical stimulation on LECs, which extend the EF-responsive cell type to cells in the lining of the lymphatic vessels. We have shown that a small steady DC EF at a physiological magnitude markedly stimulated the activation of VEGFR-3 and associated downstream signaling elements, promoting directed cell migration, cell elongation, and cell reorientation, all of which are critical to lymphangiogenesis. Notably, lymphangiogenesis is highly controlled and spatially organized. Chemical components have recently been proposed to mediate the growth of new lymphatic vessels. Our current data, therefore, propose DC EF as a novel, extracellular, and perhaps physiological cue affecting LEC behavior in culture. This biophysical cue has a series of effects that are precursors to new lymphatic vessel formation in vivo.

VEGFR-3 is a proximal element that transduces the EF and induces prolymphangiogenic responses. The downstream signaling elements of VEGFR-3 include PI3K-Akt, MAPKs, and the F-actin cytoskeleton. A previous study demonstrated that VEGFR-3 stimulation alone induces the growth and migration of isolated LECs, and the downstream signaling cascades of the VEGFR-3 mediated effects are PKC-dependent activation of Erk1/2 MAPK through wortmannin-sensitive induction of Akt phosphorylation [29]. Our finding that VEGFR-3, Akt, and Erk1/2 MAPKs are involved in EF-induced cell migration is consistent with the notion of a VEGFR-3 mediated role in LECs.

In the present study, we showed that EF enhanced the phosphorylation of p38, and inhibition of p38 MAPK led to significant inhibition of EF-mediated migration speed and elongation of HLECs, suggesting the importance of this signaling pathway in the response of LECs in EF culture. There is evidence that the p38 MAPK pathway can be activated in response to different cell types in EFs, such as embryonic stem cells and human endothelial cells [30,31]. This was inconsistent with the p38 activity induced by EF stimulation. One study showed that the p38 pathway and its downstream transcription factor AP-1 were negatively modulated by EF in lipopolysaccharide (LPS)-induced inflammatory response [32], whereas Zhao et al. reported that EF triggered the phosphorylation of p38 MAPK in keratinocytes and neutrophils [33]. These conflicting results suggest that the potential role of the MAPKs superfamily in EF stimulation may be cell type-dependent, which requires further in-depth exploration. Notably, one report showed that the total saponins of Panax notoginseng (PNS) could activate lymphangiogenesis both in vivo and in vitro by upregulating VEGF-C (a ligand specifically binding to VEGFR-3) and activating several intracellular signaling pathways including p38 MAPK [34].

Cytoskeletal reorganization underlies cell elongation, orientation, and migration induced by various stimulations [35–37]. EF-induced LEC orientation and alignment may also involve actin filament (F-actin) rearrangements because they reorient and align with the long axis of the cells after exposure to EF (Fig. S2). This result is in line with reports on EF-induced cell shape changes and cell reorientation in HUVECs and HMEC-1 [18,20].

Our previous reports have demonstrated that steady DC EF promotes angiogenesis in human vascular endothelial cells [18,21,22]. The preangiogenic response of HUVECs requires VEGF receptor activation and is mediated by the PI3K-Akt and Rho-ROCK signaling pathways [18]. In addition, EF stimulation enhances angiogenesis in vivo [15]. Therefore, EF may have therapeutic potential in ischemic diseases. Combined with our previous findings and the results of the current study, we found that EF may promote lymphangiogenesis. These properties are expected to be useful in modulating these phenomena in various diseases.

In summary, our data suggest, for the first time, that steady DC EF at a physiological magnitude may play a pro-lymphangiogenic role in an in vitro system that requires VEGFR-3 activation and is mediated through the PI3K-Akt, Erk1/2, and p38 MAPKs signaling pathways. This indicates that endogenous EF may play a role in lymphangiogenesis in vivo by stimulating the VEGFR-3 signaling pathway to induce key lymphangiogenic responses. These findings suggest that it has therapeutic potential for treating diseases related to lymphatic system impairment.

## Supplementary Material

Supplemental Material

## Data Availability

The data that support the findings of this study are available from the corresponding author (Huai Bai) upon reasonable request.
