# Supplementary figures and images for: Lymphangiogenic responses of lymphatic endothelial cells to steady direct-current electric fields
Source: Cell Adh Migr. 2023 Oct 27;17(1):1–14. doi: 10.1080/19336918.2023.2271260 (PMC10761046; doi:10.1080/19336918.2023.2271260)

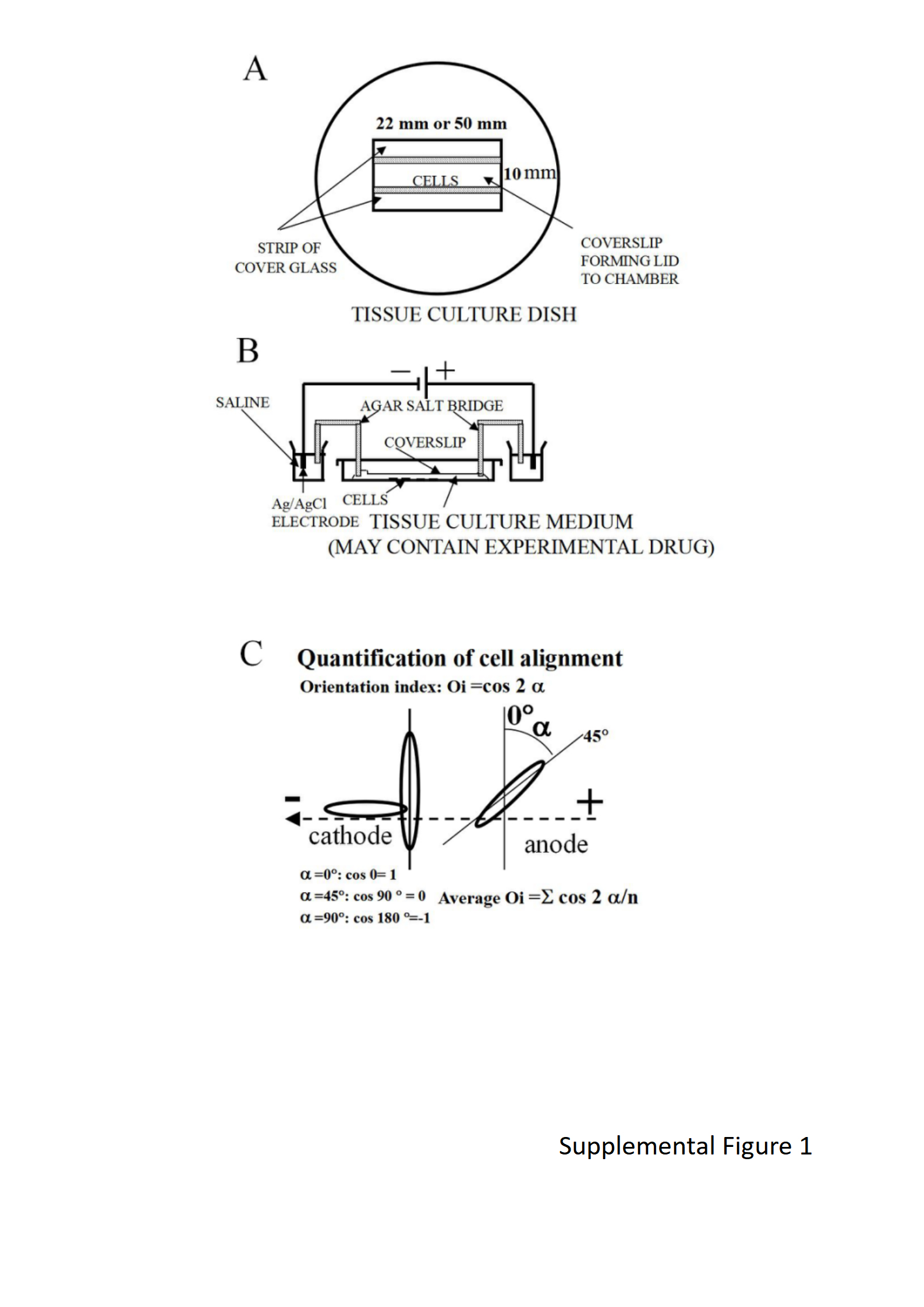

Supplement: Supplemental Material [file KCAM_A_2271260_SM2288.zip › Supplemental figure 1.jpg]

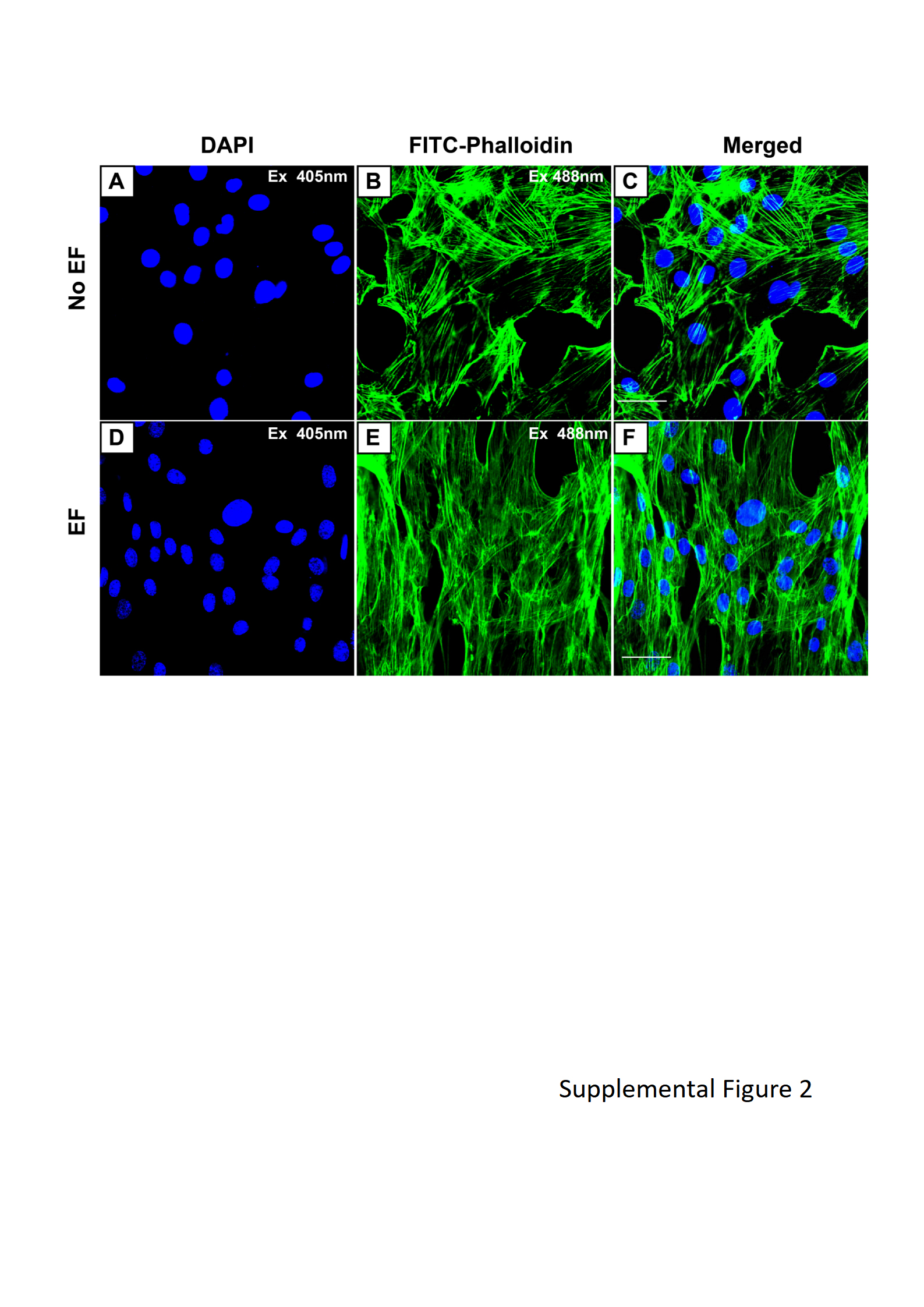

Supplement: Supplemental Material [file KCAM_A_2271260_SM2288.zip › Supplemental figure 2.jpg]

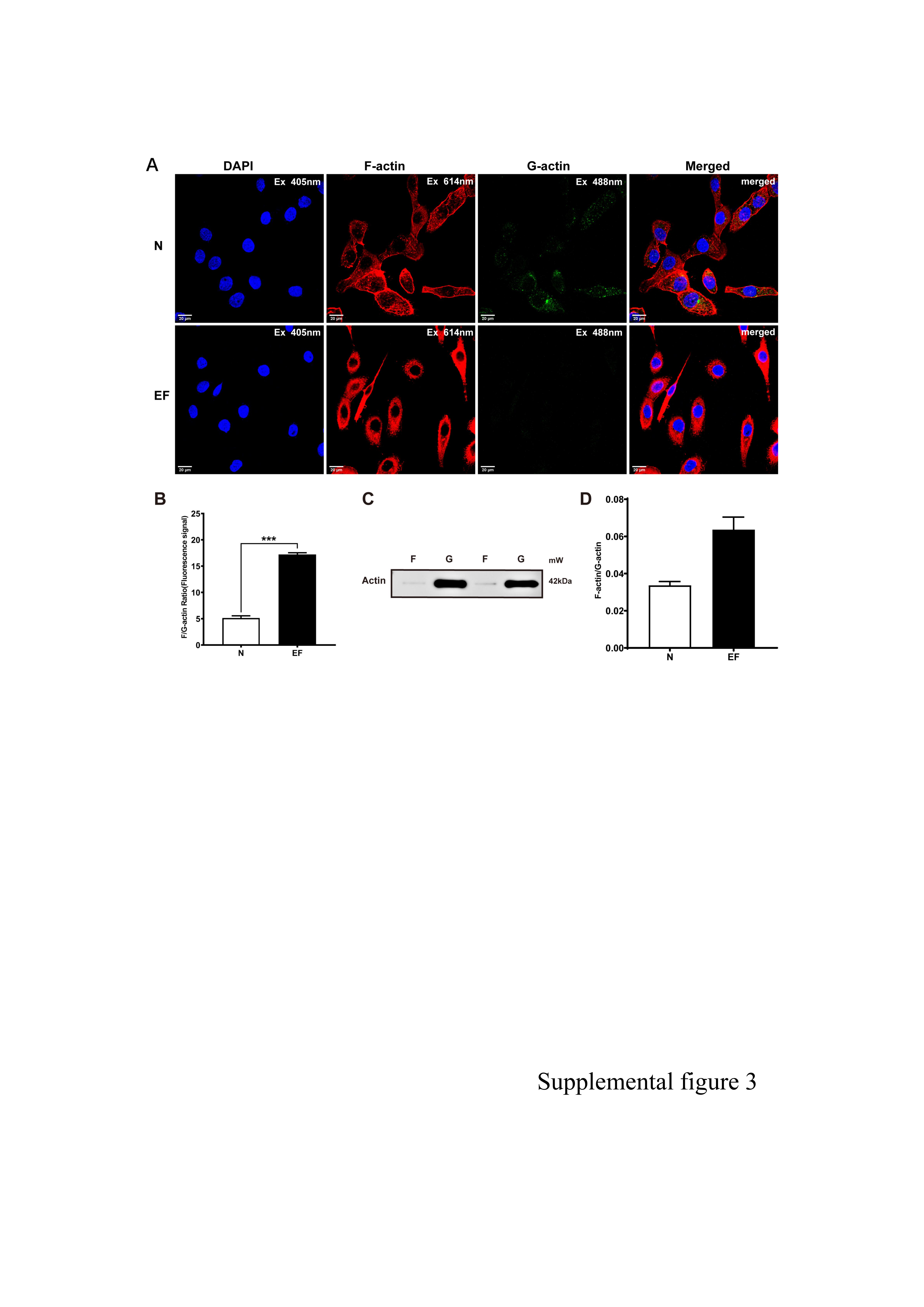

Supplement: Supplemental Material [file KCAM_A_2271260_SM2288.zip › Supplemental figure 3.jpg]

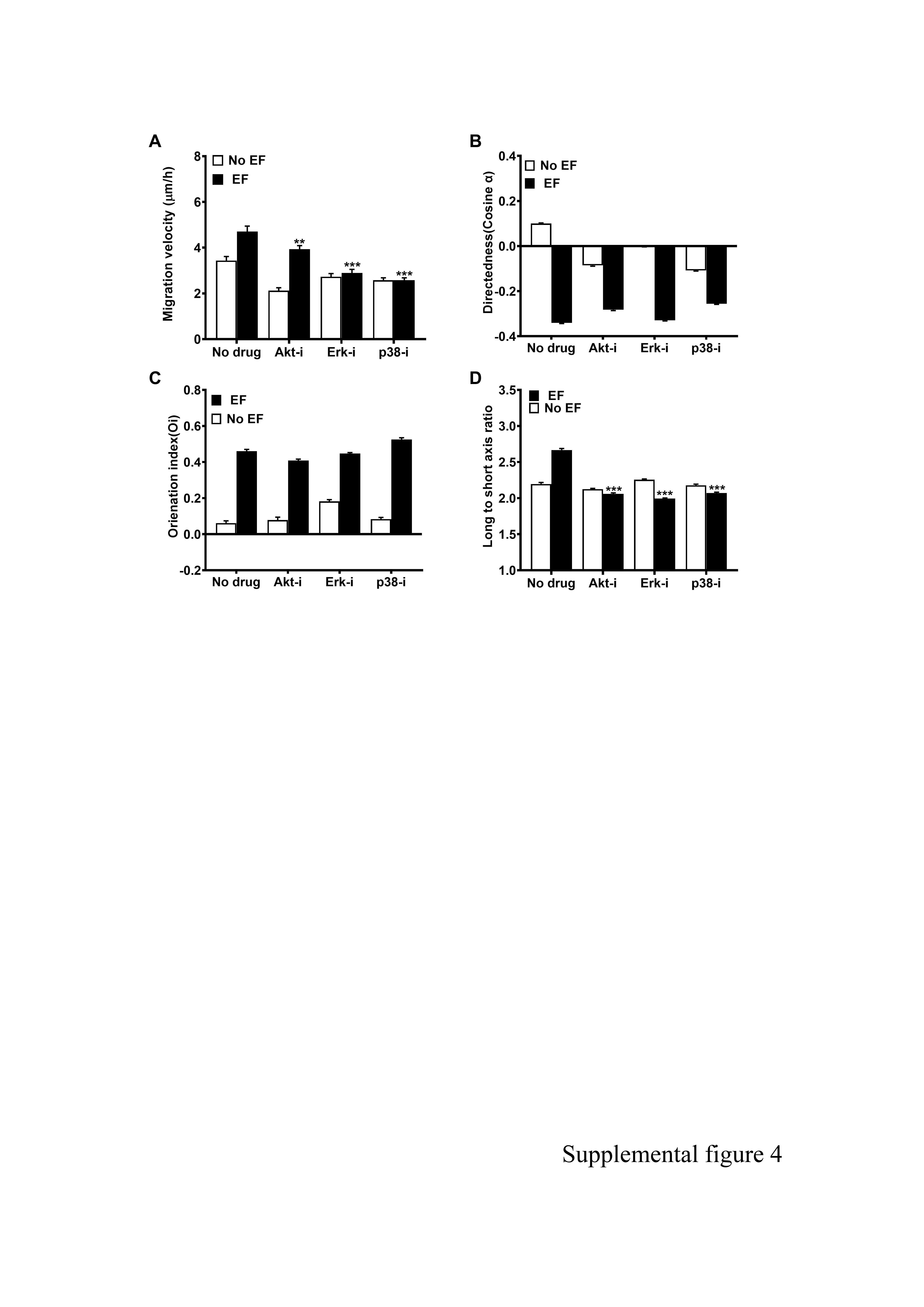

Supplement: Supplemental Material [file KCAM_A_2271260_SM2288.zip › Supplemental figure 4.jpg]

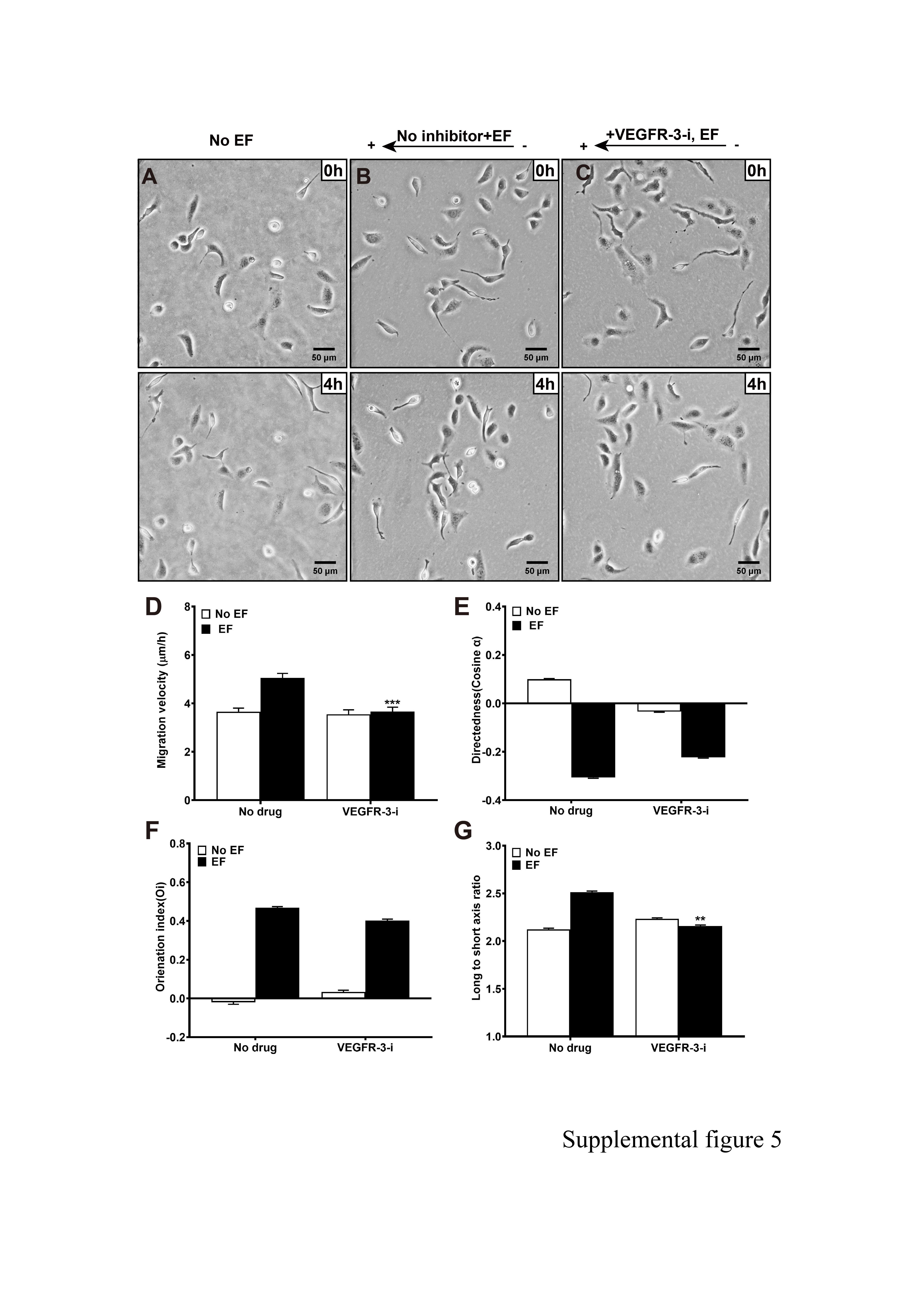

Supplement: Supplemental Material [file KCAM_A_2271260_SM2288.zip › Supplemental figure 5.jpg]

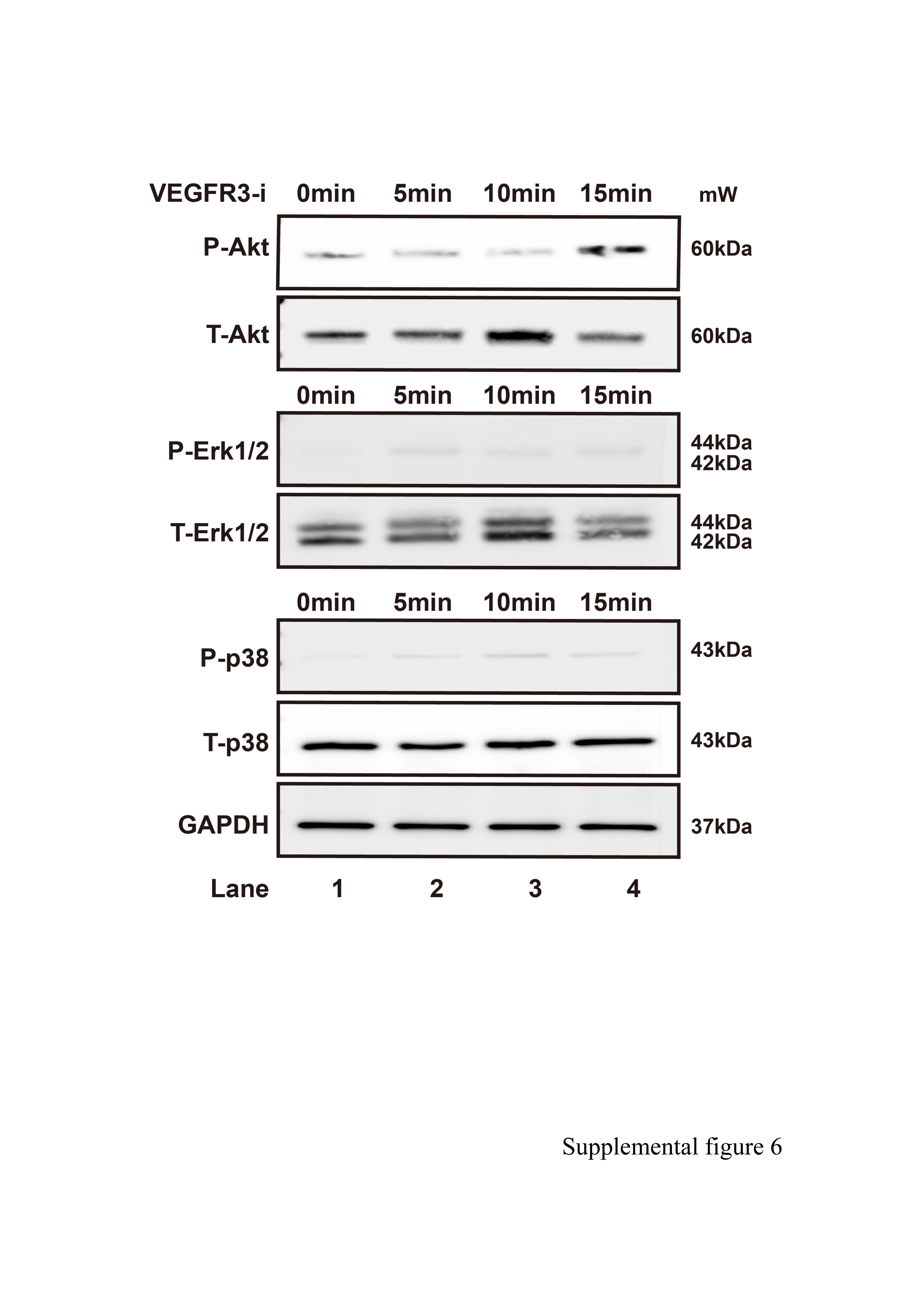

Supplement: Supplemental Material [file KCAM_A_2271260_SM2288.zip › Supplemental figure 6.jpg]

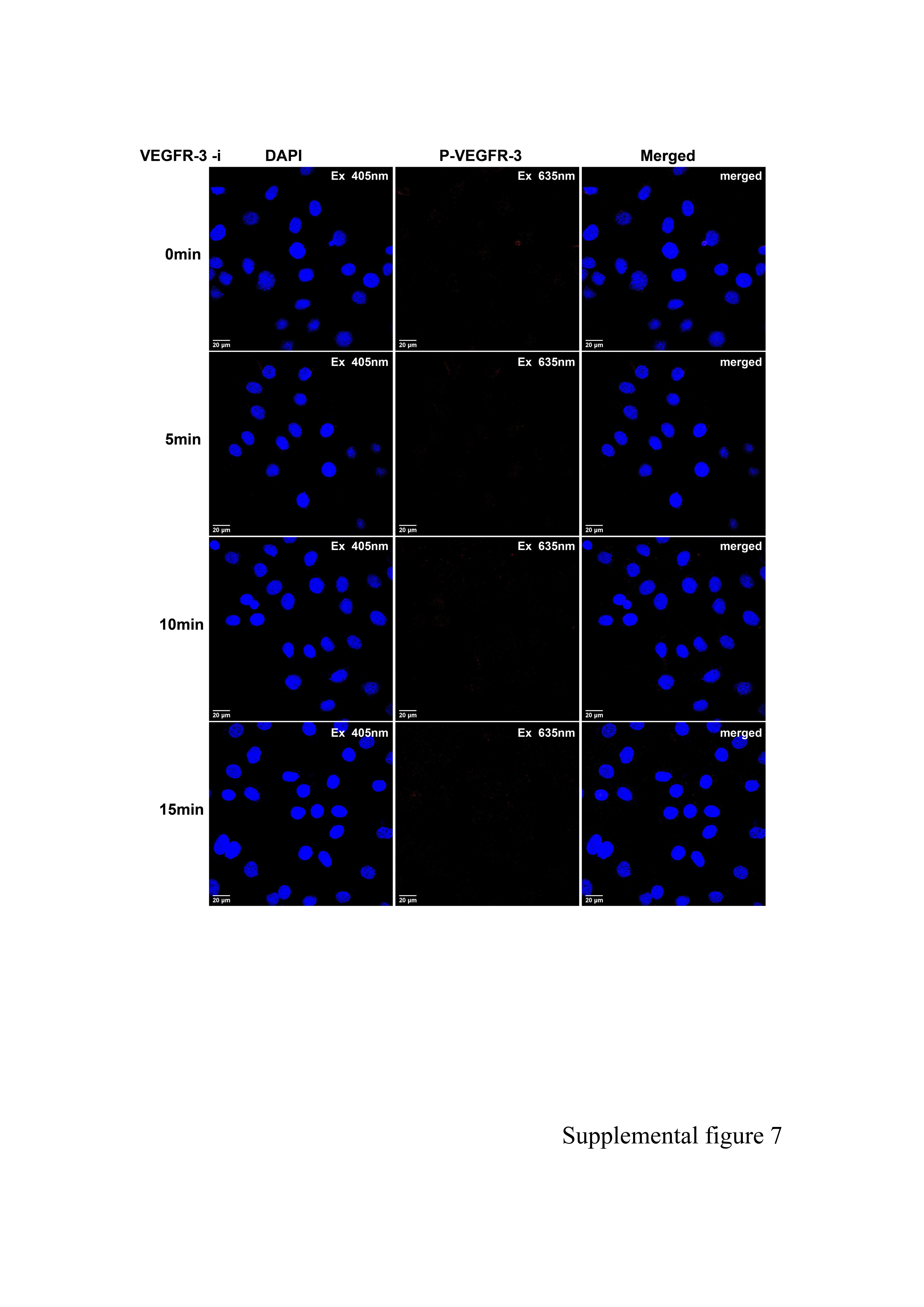

Supplement: Supplemental Material [file KCAM_A_2271260_SM2288.zip › Supplemental figure 7.jpg]

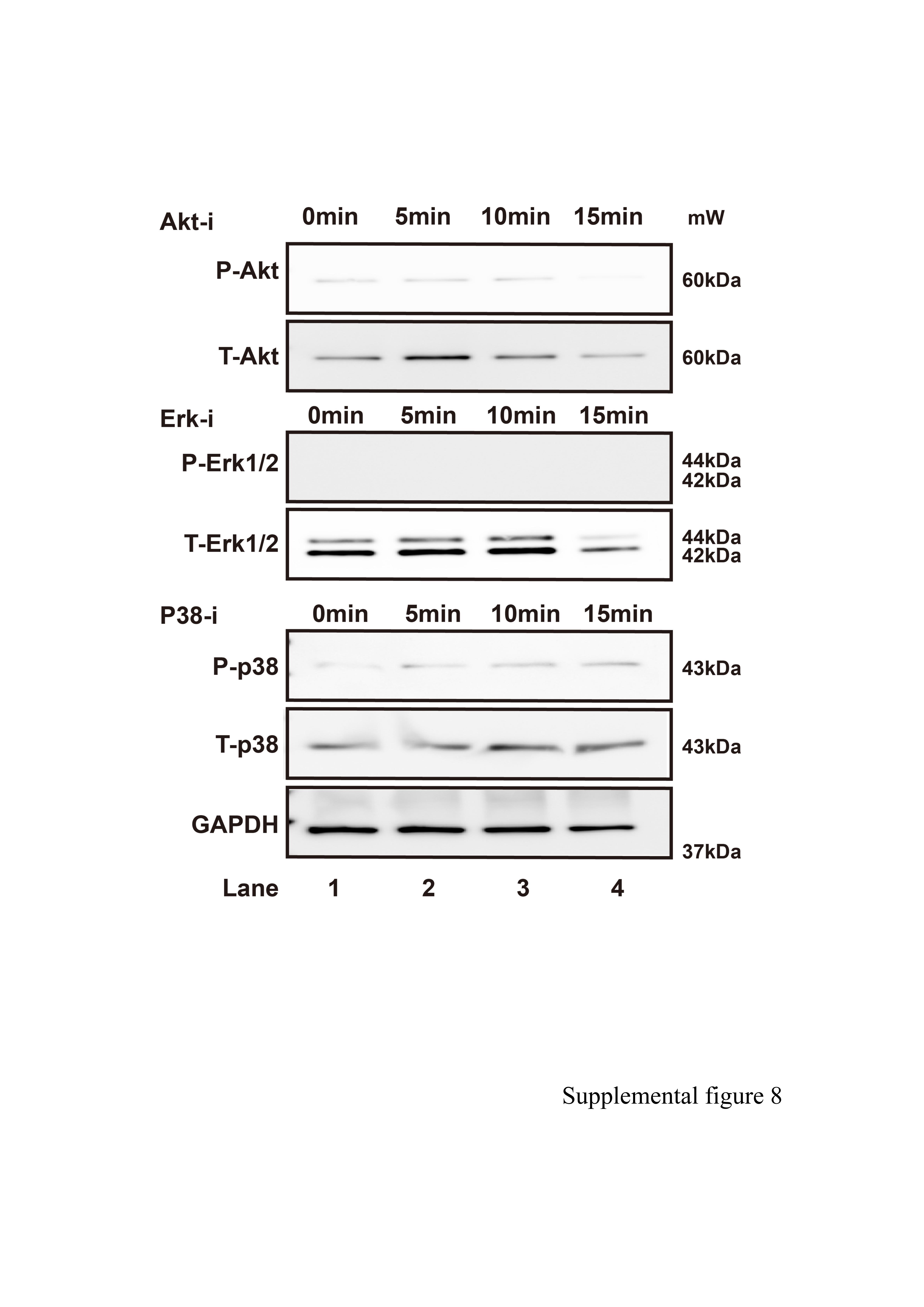

Supplement: Supplemental Material [file KCAM_A_2271260_SM2288.zip › Supplemental figure 8.jpg]
